# Evolutionary Analysis of Respiratory Burst Oxidase Homolog (RBOH) Genes in Plants and Characterization of *ZmRBOHs*

**DOI:** 10.3390/ijms24043858

**Published:** 2023-02-14

**Authors:** Haiyang Zhang, Xu Wang, An Yan, Jie Deng, Yanping Xie, Shiyuan Liu, Debin Liu, Lin He, Jianfeng Weng, Jingyu Xu

**Affiliations:** 1 College of Agriculture, Heilongjiang Bayi Agricultural University, Daqing 163319, China; 2 College of Engineering, Heilongjiang Bayi Agricultural University, Daqing 163319, China; 3 Institute of Crop Science, Chinese Academy of Agricultural Science, No. 12 Zhongguancun South Street, Haidian District, Beijing 100081, China

**Keywords:** NADPH oxidase (RBOH), evolutionary analysis, expression profiles, maize (*Zea mays*)

## Abstract

The respiratory burst oxidase homolog (RBOH), as the key producer of reactive oxygen species (ROS), plays an essential role in plant development. In this study, a bioinformatic analysis was performed on 22 plant species, and 181 RBOH homologues were identified. A typical RBOH family was identified only in terrestrial plants, and the number of RBOHs increased from non-angiosperms to angiosperms. Whole genome duplication (WGD)/segmental duplication played a key role in RBOH gene family expansion. Amino acid numbers of 181 RBOHs ranged from 98 to 1461, and the encoded proteins had molecular weights from 11.1 to 163.6 kDa, respectively. All plant RBOHs contained a conserved NADPH_Ox domain, while some of them lacked the FAD_binding_8 domain. Plant RBOHs were classified into five main subgroups by phylogenetic analysis. Most RBOH members in the same subgroup showed conservation in both motif distribution and gene structure composition. Fifteen *ZmRBOHs* were identified in maize genome and were positioned in eight maize chromosomes. A total of three pairs of orthologous genes were found in maize, including *ZmRBOH6*/*ZmRBOH8*, *ZmRBOH4*/*ZmRBOH10* and *ZmRBOH15*/*ZmRBOH2*. A Ka/Ks calculation confirmed that purifying selection was the main driving force in their evolution. ZmRBOHs had typical conserved domains and similar protein structures. *cis*-element analyses together with the expression profiles of the *ZmRBOH* genes in various tissues and stages of development suggested that ZmRBOH was involved in distinct biological processes and stress responses. Based on the RNA-Seq data and qRT-PCR analysis, the transcriptional response of *ZmRBOH* genes was examined under various abiotic stresses, and most of *ZmRBOH* genes were up-regulated by cold stress. These findings provide valuable information for further revealing the biological roles of *ZmRBOH* genes in plant development and abiotic stress responses.

## 1. Introduction

Plant NADPH oxidases (RBOHs) produce superoxide anions (·O_2_^−^) by catalyzing electron transfer from NADPH to O_2_ for the production of reactive oxygen species (ROS) [1]. In plants, ROS is known as cellular second messengers regulates a variety of aspects involving plant growth and development, as well as plant responses to environmental stresses [2,3]. ROS species are toxic when their increased accumulation exceeds a certain threshold, and may cause lipid peroxidation in cellular membranes, DNA damage, protein denaturation, carbohydrate oxidation, pigment breakdown and an impairment of enzymatic activity [4,5].

In plants, RBOHs are homologs of gp91phox, the catalytic subunit of phagocyte NOXs [6]. Previous research has suggested that six transmembrane central regions, two heme groups, cytosolic FAD (flavin adenine dinucleotide) and NADPH (nicotinamide adenine dinucleotide phosphate) binding domains are located at the C terminal of RBOH proteins [7]. Additionally, the N terminals of RBOHs feature two EF hands [7]. There are four conserved domains in typical NADPH oxidases: NADPH_Ox, Ferric_reduct, FAD_binding_8 and NAD_binding_6 [8]. In Arabidopsis, the AtRBOHs have basically similar structures, including two EF chiral structures in the N-terminal region that bind to Ca^2+^ [7]. RBOH catalyzes the transfer of electrons from NADPH (electron donor) to O_2_ (electron accepter) via NADPH-binding and FAD-binding motifs [1].

RBOHs are encoded by multiple genes. Currently, over 150 members of the RBOH family in different plant species have been identified and/or characterized in monocots, dicots and lower plants [9,10,11,12]. Ten RBOHs have been found in Arabidopsis and named *AtRbohA–J* [7], and nine RBOHs in rice [13], fourteen in *Nicotiana tabacum* [14], seventeen in soybean [10], forty-six in wheat [15], fourteen in *Brassica rapa* [16], and seven in grapes [17]. RBOHs are regarded as the center hubs in the ROS signaling network and play an essential role in stress responses and plant development [18,19,20]. It has been reported that RBOHs have a vital role in pollen development [21], pollen tube growth [22], root hair development [23], seed germination [24], plant-microorganism interactions [25] and plant immunity [26].

RBOHs play a substantial role in the response of plants to abiotic stresses such as drought, salt and extreme temperatures [18,27,28,29,30,31]. Under salt stress, H_2_O_2_ produced by superoxide dismutase (SOD) can trigger the antioxidant response in plants to overcome subsequent ROS production, and consequently mitigate the salt stress-related injuries [32]. Since NaCl is the most soluble and widespread salt, plants have evolved mechanisms to regulate its accumulation [33]. In Arabidopsis, *AtRBOHD/F* produce ROS that function as signal molecules to regulate Na^+^/K^+^ balance, resulting in salt-tolerant plants [34,35], and the double mutant *rbohD/F* was more susceptible to salt due to the ability to selectively absorb K^+^ being less efficient. NADPH oxidases can be activated by drought [36]. Drought stress increased the activities of catalase and peroxidase in Okra [37]. A drought-induced increase in *OsRBOHA* transcripts, and over-expression of *OsRBOHA,* could improve plant tolerance to water stresses in rice [38]. In plants, RBOHs and H_2_O_2_ are important components of ABA signal transduction [39]. In Arabidopsis, the NADPH oxidase inhibitor, diphenylene iodonium (DPI), inhibited ABA-induced stomatal closure, suggesting that NADPH oxidase activation is necessary [40]. 

Cold stress severely restricts plant growth and development, and is a major cause of crop loss and limiting factor to plant cultivation [41]. Plant RBOH genes are found to be transcriptionally active under low temperatures stress. Many studies have reported that RBOHs respond to cold stress. For example, cold treatment significantly increased expression of *CaRBOHA* and *CaRBOHB* in pepper [42], *GmRBOHs* in soybean [10,43] and *NtRBOHs* in tobacco [14]. Ca^2+^-dependent *AtRBOHF* mediated ROS production by *AtSRC2* was associated with cold resistance [44]. Recent research found that cold rapidly induced expression of *FvRBOHA* and *FvRBOHD* in strawberry, and *CsRBOHD*, *CsRBOHE* and *CsRBOHF* in citrus, and increased NADPH oxidase activity [11,45]. Under cold stress conditions, silencing of *CsRBOHD* in trifoliate orange resulted in cold tolerance, suggesting that the *CsRBOHD*-ROS signal pathway induces ROS scavenging enzyme genes [11].

In this study, we identified RBOH sequences from 22 plant species. The bioinformatic investigation of gene structure, evolutionary, syntenic relationships, chromosome localization, phylogeny, and conserved domains of plant RBOHs were conducted. The expression profiles of *ZmRBOH* genes were determined in different tissues and under cold stresses to decipher the roles of ZmRBOHs in maize development and stress responses. 

## 2. Results

### 2.1. Identification and Evolutionary Analysis of RBOH Family Members in Plants

An evolutionary analysis of plant RBOH genes was conducted to clarify their evolutionary history. RBOH HMMs downloaded from Pfam were used to search against the genome database of different plant species, and for homologous verification we submitted the putative RBOH homologs identified in this study to NCBI, SMART, and Pfam databases. A total of 181 candidate RBOH sequences in 22 plant species were identified initially, including chlorophyta (*Chlamydomonas reinhardtii, Volvox carteri*), bryophyta (*Physcomitrella patens, Marchantia polymorpha*), pteridophyte (*Selaginella moellendorffii*), gymnosperm (*Pinus taeda*), basal angiosperms (*Amborella trichopoda, Nymphaea colorata*), monocots (*Musa acuminata, Brachypodium distachyon, Oryza sativa, Setaria italica, Zea mays, Sorghum bicolor*), and dicots (*Vitis vinifera, Theobroma cacao, Brassica rapa, Arabidopsis thaliana, Populus trichocarpa, Glycine max, Solanum tuberosum, Solanum lycopersicum*). 

**Figure 1:**
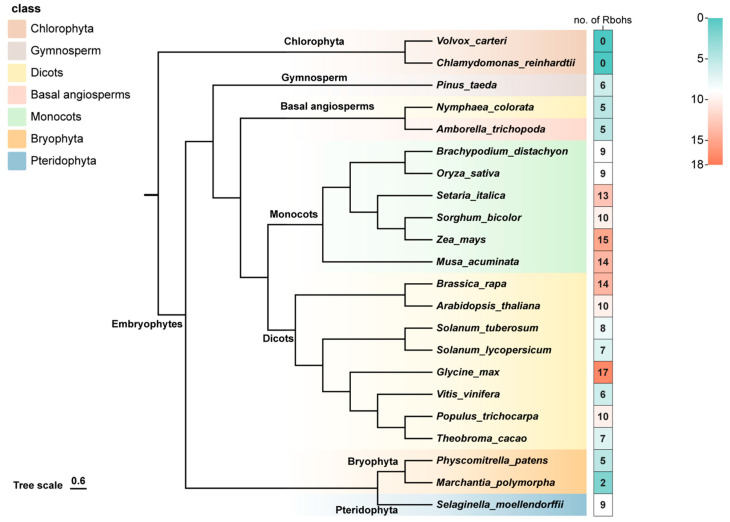
Evolutionary relationships of respiratory burst oxidase homologue (RBOH) genes among 22 plant species.

The RBOH proteins were identified in all the higher plants lineages, including angiosperm, gymnosperm, bryophyta and pteridophyte, except for two algal species selected in lower plants (Figure 1). Different plant species possess different numbers of RBOHs. No RBOH gene was found in chlorophyta, two and five RBOH genes were identified in bryophyta (*Physcomitrella patens*, *Marchantia polymorpha*), nine and six RBOH genes were discovered in pteridophyte (*Selaginella moellendorffii*) and gymnosperm (*Pinus taeda*), and there were five RBOH genes in each basal angiosperm (*Amborella trichopoda*, *Nymphaea colorata*). Increasing numbers of RBOH genes was found in higher plants: 6–17 members were detected in dicots, and 9–15 members were detected in monocots. Over all, angiosperm plants accumulated more RBOHs than non-angiosperm plants, and monocots had a higher average number of RBOHs than dicots. These results suggest that a single cell to multicellular expansion might take place in plants to expand RBOHs. Complex evolution has taken place for the typical RBOH family, which is only found in terrestrial plants.

### 2.2. The Characteristics and Conserved Domains of RBOHs in Plants

Plant RBOH encoded proteins comprise 98 to 1461 amino acids. They have a molecular weight between 11.1 and 163.6 kDa, and predicted PI values of 4.86–10.28. The subcellular localization predicted by WoLF PSORT indicated that most of the plant RBOHs were localized in the plasma membrane, eight RBOHs were localized to the nucleus, only MaRBOH9 was reside in the mitochondria and MaRBOH10 reside in the chloroplast. The detailed information of plant RBOHs (including major lineage, alternative name, accession numbers, protein length, theoretical PI, molecular weight and predicted subcellar location) are listed in Table S1.

**Figure 2:**
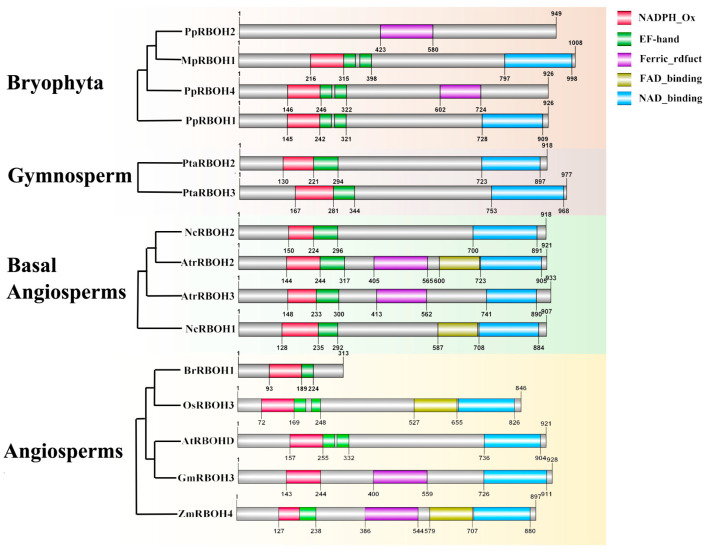
Conserved domains of the representative RBOHs in plants.

Four conservative domains were predicted in plant RBOHs using online databases in the NCBI website, including NADPH_Ox, Ferric_reduct, FAD_binding_8 and NAD_binding_6 (Figure S1). It was found that all RBOHs contained the NADPH_Ox domain, while the other four typical domains were incomplete among different species. FAD_binding_8 domain was absent in bryophyta (*Marchantia polymorpha* and *Physcomitrella patens*) and gymnosperm (*Pinus taeda*). The conserved domains of the representative RBOHs in plants are displayed in Figure 2. The results show that PpRBOH2 contained only the Ferric_reduct domain, which is similar to the common ancestor of RBOHs. PtaRBOH2 contained the NADPH_Ox domain and NAD_binding_6 domain, while PtaRBOH3 is missing the FAD_binding_8 domain (Figure 2). In two basal angiosperms (*Amborella trichopoda* and *Nymphaea colorata*) and pteridophyta (*Selaginella moellendorffii*), NcRBOH2 contained only two domains, the NADPH_Ox domain and NAD_binding_6 domain, while NcRBOH1 and AtrRBOH3 contained three domains, and AtrRBOH2 contained four typical domains. In dicots, BrRBOH1 contained only the NADPH_Ox domain, AtRBOHD contained the NAD_binding_6 domain in addition to the NADPH_Ox domain, and GmRBOH3 contained three domains but lacked the FAD_binding_8 domain. In monocots, OsRBOH3 contained three domains and ZmRBOH4 contained four typical domains. This result implies that the FAD_binding_8 domain seems to be the last obtained functional domain during the evolution of plant RBOHs. In addition, new RBOH types were identified in *Zea mays* (*ZmRBOH14*), *Solanum tuberosum* (*StRBOH3*), *Brassica rapa* (*BrRBOH1,12*) and *Musa acuminata* (*MaRBOH3,10*), which have an NADPH_Ox domain, whereas one or two other domains that typical RBOHs possess are missing.

### 2.3. Phylogenetic Analysis and Classification of RBOH Members

**Figure 3:**
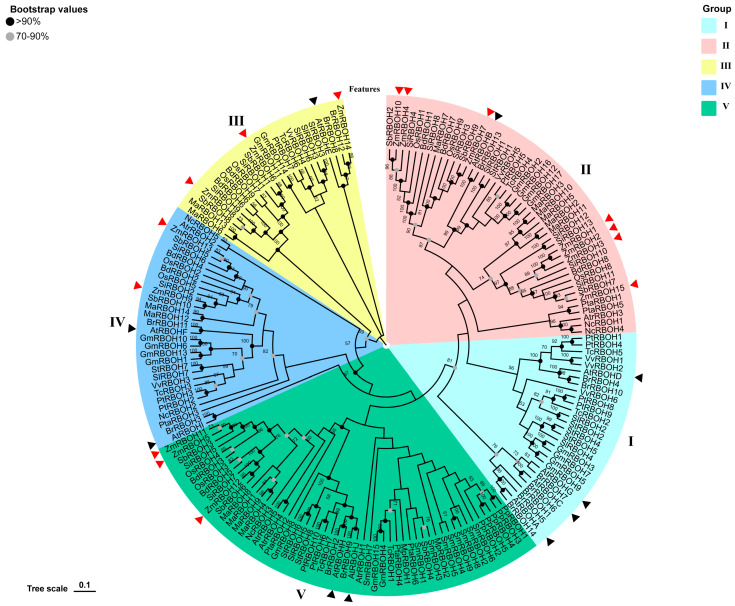
Phylogenetic relationship of plant RBOH family members. Five subgroups are indicated with I–V, respectively. Triangles in red and black color indicate *ZmRBOHs* and *AtRBOHs*, respectively.

Phylogenetic trees of the 181 RBOHs from 20 plant species were generated to explore the phylogenetic relationships among representative species of monocots, dicots, bryophytes and pteridophytes (Figure 3). One-hundred-and-eighty-one RBOHs were clustered into five subgroups based on the NJ tree, among which the highest numbers of RBOHs were 53 and 45 in subgroups Ⅴ andⅡ, followed by 30, 29 and 24 in subgroups Ⅳ, Ⅰ and Ⅲ, respectively (Figure 3 and Figure S2). Angiosperm species were classified into different clades, and the number of RBOHs was different within each clade. Ten *AtRBOHs* were dispersed across all subgroups with one to four members in each subgroup. A similar profile was observed in the distribution of RBOH in the other angiosperm species. Additionally, all members of both bryophyta (*Marchantia polymorpha*, *Physcomitrella patens*) and pteridophyta (*Selaginella moellendorffii*) were included in subgroup Ⅴ. Distribution of conserved motifs in RBOH proteins of 20 plant species are listed in Table S2. Most conserved motifs in RBOH proteins in the same subgroup were found to be conserved in both pattern distribution and composition. (Figure S3). 

Through HMMER and BLASTP searching, 15 *ZmRBOH* genes were identified from maize genomes. These candidate *ZmRBOH* genes were named as *ZmRBOH1-15*. Out of 15 *ZmRBOHs*, half of them (*ZmRBOH12, 3, 4, 7, 10* and *15*) were clustered in subgroup Ⅱ, three in subgroup Ⅴ (*ZmRBOH5, 11 and 12*) and subgroup III (*ZmRBOH6, 8* and *14*), and two in subgroup Ⅳ (*ZmRBOH9* and *13*).

### 2.4. Chromosomal Distribution and Synteny Analysis of Plant RBOH Members

**Figure 4:**
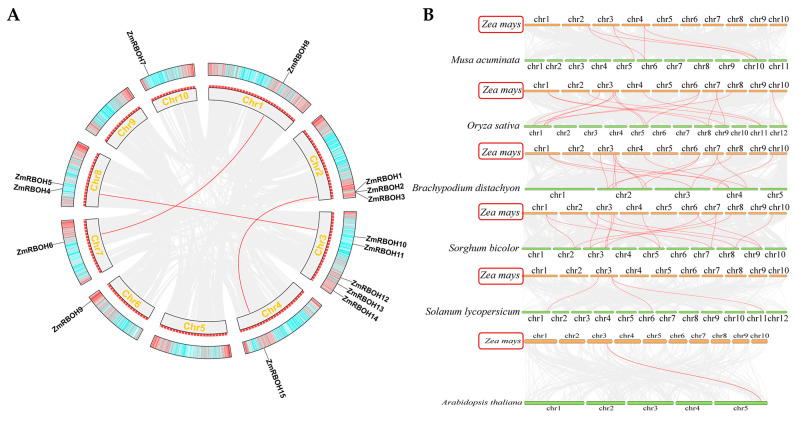
Syntenic analysis of RBOH genes. (**A**) Syntenic analysis of RBOHs and orthologous gene pairs in maize. The chromosomes are depicted as a circle. The red curves denote the syntenic regions of the *ZmRBOH* genes. Each maize chromosome was attached with 300-kb gene density information depicted by the heatmap. (**B**) The synteny of RBOH members between maize and six other species. The syntenic blocks are set as the gray background, and the syntenic RBOH members ae highlighted with red curves.

*ZmRBOH* genes are localized on chromosomes one (*ZmRBOH8*), two (*ZmRBOH1-3*), three (*ZmRBOH10-14*), four (*ZmRBOH15*), six (*ZmRBOH9*), seven (*ZmRBOH6*), eight (*ZmRBOH4* and *ZmRBOH5*) and ten (*ZmRBOH7*), respectively (Figure S4). The replication relationships were investigated among the *ZmRBOH* genes. A total of three orthologous gene pairs was found, including *ZmRBOH6*/*ZmRBOH8*, *ZmRBOH4*/*ZmRBOH10* and *ZmRBOH15*/*ZmRBOH2* (Figure 4A, Table 1). There was a common genomic origin as well as plausible functional similarity for the orthologous genes. In Figure 4A, gradient colors are shown based on the gene density of maize chromosomes, and it is apparent that the *ZmRBOH* orthologous genes tend to gather in regions with similar gene density.

Interspecific collinearity between maize and six other plant species (*Arabidopsis thaliana*, *Solanum lycopersicum*, *Musa acuminata*, *Oryza sativa*, *Brachypodium distachyon*, *Sorghum bicolor*) were analyzed using MCScanX with default parameters, and RBOH gene pairs were marked in red (Figure 4B). Among all the RBOH genes, the strength of the correlation with *ZmRBOH* genes in descending order was as follows: RBOHs from four monocots *Brachypodium distachyon* (15), *Sorghum bicolor* (15), *Oryza sativa* (14) and *Musa acuminata* (5), and RBOHs from two dicots *Solanum lycopersicum* (3) and *Arabidopsis thaliana* (1). It turns out that maize and other monocots have more orthologous genes than dicots, which is consistent with their evolutionary relationships. It is worth noting that the frequency of homologous genes on chromosome 3 is the highest in both monocots and dicots, which indicates that this site may be an important site for RBOH gene selection during evolution. 

Gene duplication occurs in five types: singleton, dispersed, proximal, tandem, and whole genome duplication (WGD)/segmental duplication. In this study, numbers of genes from different duplication events were detected in the RBOHs of five monocots by running the MCScanX package. Among the five duplication types, we found that WGD/segmental and dispersed played a key role in RBOH gene family expansion in all five monocots and proximal was detected only in maize. The percentage of RBOHs that underwent WGD/segmental duplication was 40.0, 85.7, 66.7, 66.7, and 40.0% for *Zea mays*, *Musa acuminata*, *Oryza sativa*, *Brachypodium distachyon* and *Sorghum bicolor*, respectively. The percentage of RBOHs that underwent dispersed was 46.7, 14.3, 33.3, 33.3 and 60.0% for *Zea mays*, *Musa acuminata*, *Oryza sativa*, *Brachypodium distachyon* and *Sorghum bicolor*, respectively (Table 1). To assess the evolutionary rates among these gene-pairs, the Ka (non-synonymous substitutions) to Ks (synonymous substitutions) ratios of homologs in the *ZmRBOH* gene family were calculated (Table S3). It is noteworthy that all Ka/Ks ratios between gene-pairs with collinearity relationship were less than 1, implying that these genes have undergone strong purifying selection pressures to different extents during evolution.

### 2.5. Analysis of Domain Composition, Gene Structure, and Conserved Motifs of ZmRBOH Genes

**Figure 5:**
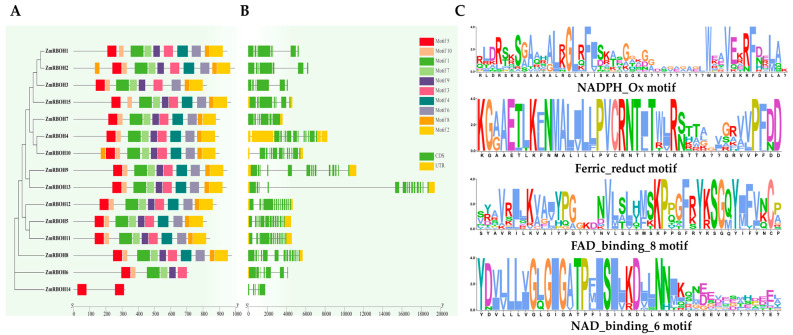
Evolutionary relationships, conserved motifs, and gene structures of *ZmRBOH* genes. The phylogenetic tree was constructed using MEGA-X by the Neighbour-Joining (NJ) method. (**A**) Conserved motifs in ZmRboh proteins. (**B**) Gene structures of the *ZmRBOH* genes. Exons, introns, and untranslated regions (UTRs) are indicated by yellow rectangles, gray lines, and green rectangles, respectively. (**C**) Sequence-logos of conserved motifs. The bit score indicates content of each position in the amino acid sequence.

The NJ tree, gene structure and motif of 15 *ZmRBOH* sequences were mapped by TBtools. The conserved domains of the ZmRBOHs were illustrated by MEME-motif scanning. As shown in Figure 5A, ten MEME-motifs were collected (Motifs 1 to 10). In accordance with conserved domains, ZmRBOHs within each subgroup displayed similar motif distribution. In general, most ZmRBOHs showed regular motifs in their specific clades. Motif 5 and motif 10 were found in the NADPH_Ox domain. Ferric_reduct domain dominated motifs 1 and 7. The FAD_binding_8 domain contained motif 4, and the NAD-binding domain was formed by motif 6. Sequence-logos of domain-motifs are displayed in Figure 5C. Detailed information of the distribution of conserved motifs in ZmRBOH proteins is listed in Table S2. As shown in Figure 5B, diverse gene structures was found among different types of *ZmRBOH* genes, whereas there was a high degree of conservation in each group. The number of exons in 8 out of 15 *ZmRBOH* genes was between 12–14, whereas other *ZmRBOH* genes contained 5-8 exons, respectively. Based on sequence alignment, it was found that conserved motif NADPH_Ox was present in all ZmRBOHs. All four characteristic motifs: NADPH_Ox, Ferric_reduct, FAD_binding_8 and NAD_binding_6, were present in ZmRBOH4, ZmRBOH5 and ZmRBOH11. ZmRBOH14 had only one (NADPH_Ox) of the RBOH-characteristic motifs, which was different from other ZmRBOHs. ZmRBOH6 and ZmRBOH8 had two conserved motifs (NADPH_Ox and Ferric_reduct in ZmRBOH6, and NADPH_Ox and NAD_binding_6 in ZmRBOH8). There was no Ferric_reduct domain in ZmRBOH12, and the FAD_binding_8 domain was missing in the remaining 8 ZmRBOH proteins (Figure 5C and Figure S1). 

### 2.6. Expression Profiles of ZmRBOH Genes in Different Tissues and Developmental Stages

**Figure 6:**
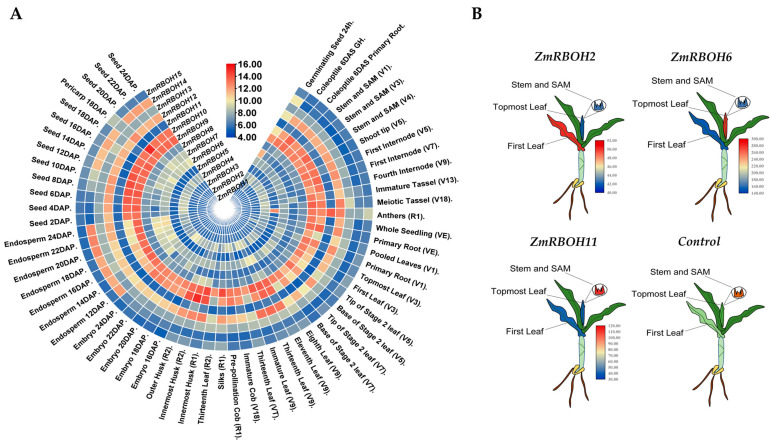
Differential expression of *ZmRBOH* genes in different tissues and developmental stages of maize. (**A**) The integrative heatmap shows the different expressed *ZmRBOH* genes in different tissues and developmental stages of maize. (**B**) Heatmaps of phenotype simulation of different expressed representative *ZmRBOH* genes in V2 stage of maize. Colors represent log2-fold change comparing relative expression, color scale is provided. Red: high expression level; blue: low expression level.

An integrative heatmap was created to illustrate the *ZmRBOH* gene expression profiles in different tissues and developmental stages of maize (Figure 6A). The transcripts of *ZmRBOH9*, *ZmRBOH10* and *ZmRBOH12* were detected in all tissues and developmental stages with a high transcriptional level. The expression of *ZmRBOH4, ZmRBOH6* and *ZmRBOH13* were at an intermediate level in all tissues and developmental stages. *ZmRBOH1-3* genes were expressed at relative lower levels in various tissues and developmental stages of maize. *ZmRBOH9* had a higher expression level during seed development in reproductive stage. *ZmRBOH7 and ZmRBOH14* may play a role in seed development as they display higher expression levels in the seed and endosperm. The transcripts of *ZmRBOH5* and *ZmRBOH11* were mainly shown in the anther. To explore the potential roles of *ZmRBOH* genes in various biological processes, we analyzed the *cis*-elements in the promoter regions of the *ZmRBOH* genes (Figure S5). The *cis*-element analyses further supported the potential roles of the identified *ZmRBOH* genes in regulating maize developments and responses to various biological processes. Overall, our findings revealed the diverse expression profiles of *ZmRBOH* genes in different tissues and developmental stages, which might imply differential roles of the *ZmRBOH* genes in maize. 

To better visualize the specific expression of *ZmRBOHs*, three representative *ZmRBOH* genes were selected to create a heatmap of phenotype simulation of V2 stage maize seedlings using TBtools (Figure 6B). The results showed that *ZmRBOH2* exhibited higher expression levels in first leaf, *ZmRBOH6* exhibited higher expression levels in topmost leaf and *ZmRBOH11* exhibited higher expression levels in stem and shoot apical meristem (SAM). Further normalization and integration of the 15 *ZmRBOH* gene expression data were performed to display the expression profiles of the *ZmRBOH* genes at the V2 stage of maize development (Figure S6). *ZmRBOH1, 2, 3, 7, 14* and *15* mainly expressed in first leaf, *ZmRBOH4, 5, 8, 10, 11* and 12 showed relatively high expression in stem and SAM, *ZmRBOH6, 9, 13* and *14* highly expressed in topmost leaf in V2 stage of maize. 

### 2.7. Expression Profiles of ZmRBOH Genes in Response to Abiotic Stress Treatment

**Figure 7:**
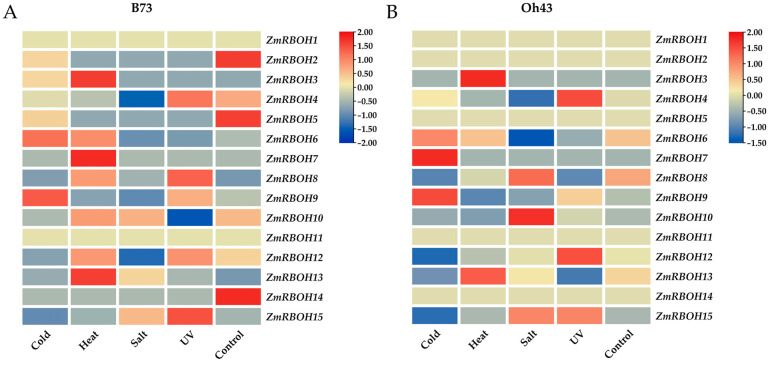
Expression profiles of *ZmRBOH* genes under abiotic stress. (**A**) Expression of *ZmRBOH* genes in maize inbred line B73. (**B**) Expression of *ZmRBOH* genes in maize inbred line Oh43. Colors represent log2-fold change comparing relative expression, color scale is provided. Red: high expression level; blue: low expression level.

To uncover the roles of *ZmRBOH* genes in abiotic stress responses, their expression data from published transcriptome of maize under various abiotic stress treatments were analyzed, including heat, cold, salt and UV (Figure 7A,B). Two maize inbred lines B73 and Oh34 were investigated. Under cold treatment, the expression of three *ZmRBOH* genes shared similar trend in both inbred lines, among which *ZmRBOH9* was up-regulated while *ZmRBOH12* and *ZmRBOH15* were down-regulated. Under heat treatment, differential expression of six *ZmRBOH* genes was found in B73, while only one gene was differentially expressed in OH43. Among them, the expression of *ZmRBOH13* gene increased significantly in both maize inbred lines. The expression of *ZmRBOH6*, *ZmRBOH8* and *ZmRBOH12* increased significantly, while the expression of *ZmRBOH9* and *ZmRBOH4* decreased significantly in B73 under heat stress. However, the expressions of these genes were not significantly changed in OH43. Under salt (NaCl) treatment, a number of *ZmRBOH* genes were down-regulated, but no genes were significantly up-regulated. In Oh43, the expression of *ZmRBOH15* increased significantly, and the expression of *ZmRBOH9* and *ZmRBOH4* decreased significantly. Six *ZmRBOH* genes were differentially expressed in B73 and Oh43. Under ultraviolet (UV) treatment, their expression profiles were different. The expression of *ZmRBOH9*, *ZmRBOH12*, *ZmRBOH4* and *ZmRBOH15* genes in the two inbred lines was significantly increased. The expression *ZmRBOH13* and *ZmRBOH8* was significantly up-regulated in B73, but down-regulated in Oh43.

### 2.8. qRT-PCR Analysis of the Expression of ZmRBOH Genes under Cold Stress

**Figure 8:**
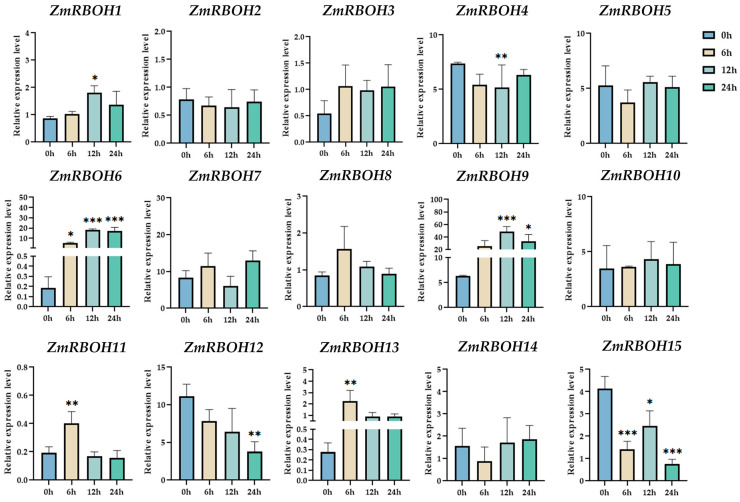
Quantitative RT-PCR analyses of *ZmRBOH* genes under cold stress. The results of quantitative RT-PCR were normalized to the *ZmEF1α* and *ZmActin* housekeeping gene. The error bars indicate the standard deviations and the values in plots corresponding to the mean ± standard deviation (SD) of three independent biological replicates (* *p* < 0.05; ** *p* < 0.01; *** *p* < 0.001).

In order to further understand how *ZmRBOH* genes respond to cold stress, the expression of 15 *ZmRBOH* genes under low temperature treatment at different time points were determined by qRT-PCR in B73 inbred lines (Figure 8). The results show that the expression of *ZmRBOH* genes were temporally regulated in response to cold. In the early stages of cold treatment, the expression levels of *ZmRBOH11* and *ZmRBOH13* were significantly increased at the 6 h time point. *ZmRBOH4* was significantly down-regulated and *ZmRBOH1* was significantly up-regulated at the 12 h time point. The expression of the *ZmRBOH12* gene was significantly down-regulated after a 24 h cold treatment. There was a significant increase in transcript abundance of *ZmRBOH6* and *ZmRBOH9* at 24 h, while *ZmRBOH15* expression was significantly down-regulated at the same time point. This suggests a quick and active responses of *ZmRBOHs* to cold stress.

## 3. Discussion

RBOHs have been discovered in animals, higher plants, and fungi since the first NADPH oxidase was discovered in human phagocytic cells [15,46,47]. In plants, typical RBOHs were identified only in terrestrial plants. Liu et al.’s research shows that there is no RBOH genes in chlorophyta (*Chlamydomonas reinhardtii*, *Volvo carteri*) [31]. However, four RBOH candidates were identified from *Chlamydomonas reinhardtii* in a recent study [48]. Previously, several RBOH homologs were identified from green algae and red algae, but assigned to another gene family (FROs), considering they did not have the NADPH_Ox domain [49,50].

**Figure 9:**
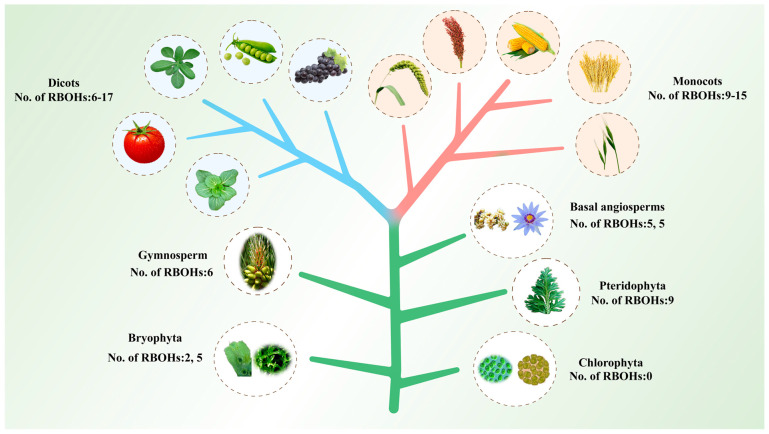
Model of occurrence of RBOHs in major plant species.

The RBOH family has a complex evolution. Liu et al. selected 17 halophyte and 21 glycophyte angiosperms for the study of RBOH family, and nine members were identified in *Selaginella moellendorffii*, which is extremely tolerant to dehydration [31]. Liu et al.’s research found that tolerant species of salinity stress have fewer RBOH genes, and salinity tolerance evolved in plants may be associated with the decline in RBOH members [31]. Chang et al. found that ancient RBOHs harboring only the Ferric_reduct domain obtained another important domain, NADPH_Ox, and were converted into the typical RBOHs in the plant [47]. A primary evolution model of RBOHs from bacteria, fungi, animal and plants has been constructed, while the evolutionary process of RBOH is not well illustrated in plants. In this study, 181 RBOH homologues from 22 plant species were identified and analyzed. Our results revealed that RBOHs only exist in land plants, and their major structural domains are similar to the previous identified RBOHs. Over the course of evolution, RBOH genes have become more and more numerous, and monocot species have a higher average number of RBOHs than dicots (Figure 9). During the evolution from gymnosperms to angiosperms, the genetic structure became more and more complicated. Plants with the NADPH_Ox domain live exclusively on land, suggesting that this domain contributes specifically to terrestrial adaptation. A long-term evolution history of RBOHs in plants clearly demonstrates their functional divergence.

In this study, most plant RBOH proteins were predicted to be localized to the plasma membrane, suggesting that membrane-bound RBOHs are the main reactive oxygen species (ROS) generators involved in plant development. Eight RBOHs were localized to the nucleus, only *MaRBOH9* was indicated to reside in the mitochondria, and *MaRBOH10* was indicated to reside in the chloroplast. Hu et al. analyzed four RBOH genes using a transient transformation system after cloning the cDNA sequences to verify the subcellular localization of *TaNOX7-3AS*, *TaNOX10-5BL*, *TaFRO4-2BL*, and *TaNOX-like4*, and the results showed they were all located with the plasma membrane [15]. In grape, several RBOHs encoded by *VvRBOHA*, *VvRBOHC1* and *VvRBOHD* were predicted to be localized in the plasma membrane, and several RBOHs were predicted to be located within the chloroplast thylakoid membrane, including *VvRBOHB*, *VvRBOHC2*, *VvRBOHE* and *VvRBOHH* [17].

Gene duplication is a major effect of whole genome duplication (WGD), which doubles the entire genome. In order to improve species diversity and environmental resilience, WGDs were thought to have occurred during the evolution of plants [51,52]. There have been several gene duplication events which drove the evolution of RBOH gene families, especially WGDs [42]. Li et al. suggest that *BrRBOH* gene expansion might have been facilitated by WGDs, which led to structural and functional novelty that enabled tolerance to abiotic and biotic stresses [16]. The results of synteny analysis in upland cotton revealed that during the evolution of the *GhRBOH* gene family, most of the duplicate genes originated from WGD or segmental duplications, which were the major factor in its expansion [48]. In this study, numbers of genes from different duplication events were detected in the RBOHs of five monocots. We found that WGDs and dispersed type duplication events played a key role in RBOH gene family expansion in monocots. In this study, 56 syntenic gene pairs were identified among 14 *ZmRBOHs* and *RBOHs* from other plant species, including 3 and 53 pairs with intraspecies and interspecies collinearity, respectively. In light of the fact that the collinearity relationships between *BrRBOH* and other plant RBOHs were only found in dicots, and the number of collinear relationships among *ZmRBOHs* and other *RBOHs* is bigger in monocots than in dicots, there is a possibility that duplication activities occurred after monocots and dicots diverged.

Plant RBOHs are involved in multiple cellular functions via ROS production and signaling [53,54]. By producing ROS, RBOH maintains pollen tube growth at normal rates [22,55]. *AtRBOHH* and *AtRBOHJ* genes are expressed in pollen, and contribute to the positive feedback regulation that maintains growth of pollen tubes [6,54,56,57]. In this study, *ZmRBOH5* and *ZmRBOH11* were highly expressed in anthers, and may have special biological functions in pollen development. According to Muller *et al*., *AtRbohB* is essential for seed ripening and germination, and inhibition of it by diphenylene iodonium (DPI) leads to a delay in seed germination of *Arabidopsis* [58]. In barley, superoxide anions (·O2^−^) produced by NADPH oxidase regulate seed germination and seedling growth [59]. In this study, *ZmRBOH9* was consistently highly expressed during seed formation, and *ZmRBOH7* and *ZmRBOH14* genes were also highly expressed in seeds and endosperm, suggesting a vital role of *ZmRBOHs* in seed germination.

RBOHs are notably responsive to various abiotic stresses, especially cold [60]. The transcription of *AtRBOHA* was up-regulated greatly, while *AtRBOHD*, *E*, *I*, and *H* were down-regulated by cold stress [44]. Almost all the *OsRBOHs* were up-regulated in both shoots and roots at the 12 h time point under cold stress [47]. Under cold stress treatment, a total of seven *GhRBOHs* were down-regulated at early time points and up-regulated after experiencing a longer cold treatment period [48]. Under cold stress, the expression of *FvRBOHA* and *FvRBOHD* was rapidly increased, followed by an increase in NADPH oxidase activity, leading to ·O2^−^ accumulation and activation of antioxidant reaction [45]. In this study, the transcription of *ZmRBOHs* was quickly induced by cold stress at all-time points, indicating their potential important roles in response to cold stress.

In summary, 181 RBOHs were identified from 22 plant species in this study, including 15 *ZmRBOH* genes. Plant RBOH genes underwent an evident evolution, and an important role was played by WGD in the expansion of the RBOH family. *ZmRBOH* genes were found to be highly expressed in seed developmental stages and anther tissues. *ZmRBOH* genes responded actively to abiotic stress, especially cold stress. Further functional characterization of RBOHs is required to obtain more direct evidence supporting the critical roles of the RBOH genes in development and stress responses in maize and other plants.

## 4. Materials and Methods

### 4.1. Data Retrieval and Identification of RBOH Genes

RBOH family members were identified from 22 sequenced genomes. These genomes were collected from Phytozome website [61] and PlantGenIE website. The Hidden Markov Model (HMM) profile [62] of the respiratory burst NADPH oxidase domain (PF08414) was downloaded from the pfam website [63] as queries. HMMER was used to identify the members of the RBOH superfamily from the downloaded protein sequences of 22 species. RBOH genes were searched against NCBI website; all sequences were downloaded and used as the database for RBOH screening. The local BLASTp (E-value < 1 × 10^−5^) was used to compare the RBOH protein sequences of 22 plant species with the sequences of RBOH gene family in Arabidopsis to check whether there were missing RBOH family members. After validation by the online SMART tool [64], multiple sequence alignment was performed in MEGAX using MAFFT software [65] to eliminate sequences of different transcripts. The WoLF PSORT website was used to search for the subcellular localization. In addition, pI and protein molecular mass of the RBOH gene family were identified from the ExPasy website (http://web.expasy.org/protparam/, accessed on 15 December 2022) [66].

### 4.2. Phylogenetics and Classifications of RBOH Members

The phylogenetic analysis was constructed via OrthoFinder [67] based on the single-copy genes identified in all the plant genomes. Multiple-sequence Alignment was conducted using MAFFT software [65], and a 1000-replication boot strap replicated the phylogenetic tree using MEGA X by neighbor-joining algorithm [68].

### 4.3. Chromosomal Location, Duplication and Synteny Analyses of ZmRBOH Genes

The chromosomal locations of *ZmRBOH* genes were depicted with TBtools based on the maize genome annotation file. Annotations of the maize genome were used to extract 300-kb hereditary interval gene densities that were further transformed into gradient colored heatmaps on each maize chromosome. Synteny analysis was carried out using the program MCScanX [69], and the parameters were set to default: Match score, 50; Match size, 5; GAP penalty, –1; Overlap window, 5; E-value, 1 × 10^−5^; Max GAPs, 25. Further, MCScanX was also used to identify WGD/segmental, tandem, proximal and dispersed duplication events in the RBOH family [69]. Syntenic graphs of multiple species were generated by TBtools [70]. Non-synonymous substitutions (ka), synonymous substitutions (ks) and Ka/Ks ratios of *ZmRBOHs* orthologous gene pairs were calculated using Ka/Ks-Calculator 2.0 [71].

### 4.4. Gene Structure, Protein Motif and Conserved Domain Analysis

The motif analysis of RBOH proteins was carried out using MEME [72]. The optimized parameters were as follows: number of repetitions, any; the maximum number of motifs, 10; and the optimum width of each motif, between 6 and 200 residues. The conservative domains of RBOHs were predicted through NCBI. Based on the genome annotation data, the gene structures of *ZmRBOHs* were determined by TBtools [73]. The Seq Logos associated with the MEME-motifs, gene structure, protein motif and conserved domain were illustrated using TBtools [73].

### 4.5. Expression Profiles of ZmRBOH Genes

RNA-seq data of different maize tissues and developmental stages were downloaded from the Maize Efp [74] database for *ZmRBOH* genes. The correlation heatmap was generated using the expression correlation matrix by TBtools. Based on the extractions of the *ZmRBOH* genes upstream 2000 bp sequences with TBtools, the *cis*-elements in gene promoter regions were explored with PlantCARE online tools (http://bioinformatics.psb.ugent.be/webtools/plantcare/html/, accessed on 15 December 2022) [75]. To better exhibit gene expression profiles in tissues, a heatmap of phenotype simulation was illustrated using TBtools. The data of transcriptome of 2-week-old maize seedlings of B73 and Oh43 under various stresses from NCBI database with three replicates was downloaded to analyze the expression profiles of *ZmRBOH* genes [76]. The stress treatment was conducted at low temperature (4 °C) for 16 h, high temperature (50 °C) treatment for 4 h, salt (300 mmol/L NaCl) treatment for 20 h and UV treatment for 2 h.

### 4.6. Plant Materials and Stress Treatments

The maize seedlings were grown in a growth chamber in vermiculite and soil (1:1, volume/volume) at 25 °C and a 16 h/8 h (light/dark) photoperiod. A low temperature treatment was conducted by placing two-week-old maize seedlings in an incubator at 4 °C. The leaf samples were collected at 0, 6, 12 and 24 h after treatment, and control samples were collected from plants under room temperature. At least three replicates were applied for each sample.

### 4.7. Quantitative RT-PCR Analysis

Total RNA was extracted using the total RNA extract reagent (Coolaber, BeiJing, China). Using Rever Tra Ace qPCR RT Master Mix (TOYOBO, Osaka, Japan), first-strand cDNA was synthesized from one microgram of RNA from each sample. A 2×SYBR Green qPCR Mix System (Coolaber, BeiJing, China) was used for real-time quantitative RT-PCR (qRT-PCR). *ZmEF1α* and *ZmACTIN* were used for standardization of target genes. The results were analyzed using the 2^−ΔΔct^ method with three replicates in three independent biological replicates [77]. Gene specific primers of *ZmRBOHs* used for qRT-PCR are shown in Table S4.

### 4.8. Statistical Analysis

A Graphpad Prism 8 program was used for Student’s *t*-test, with a significance for difference at a *p*-value cut-off of 0.05. In SUBthe column diagrams, error bars represent standard deviations (SD) from independent biological replications.

## Figures and Tables

**Table 1:** Numbers of RBOH genes from different duplication events in five monocots.

Species	Identified RBOH Genes	No. of Gene Pairs	Duplication Events				
			Singleton	WGD	Tandem	Proximal	Dispersed
*Zea mays*	15	3	0	6 (40.0%)	0	2 (13.3%)	7 (46.7%)
*Musa acuminata*	14	9	0	12 (85.7%)	0	0	2 (14.3%)
*Oryza sativa*	9	3	0	6 (66.7%)	0	0	3 (33.3%)
*Brachypodium distachyon*	9	3	0	6 (66.7%)	0	0	3 (33.3%)
*Sorghum bicolor*	10	2	0	4 (40.0%)	0	0	6 (60.0%)

## Data Availability

Not applicable.

## Supplementary Materials

The supporting information can be downloaded at: https://www.mdpi.com/article/10.3390/ijms24043858/s1.
