# Supplementary figures and images for: Evolutionary Analysis of Respiratory Burst Oxidase Homolog (RBOH) Genes in Plants and Characterization of ZmRBOHs
Source: Int J Mol Sci. 2023 Feb 14;24(4):3858. doi: 10.3390/ijms24043858 (PMC9965149; doi:10.3390/ijms24043858)

**Figure S1.** Conserved domains in RBOH proteins of 20 species.

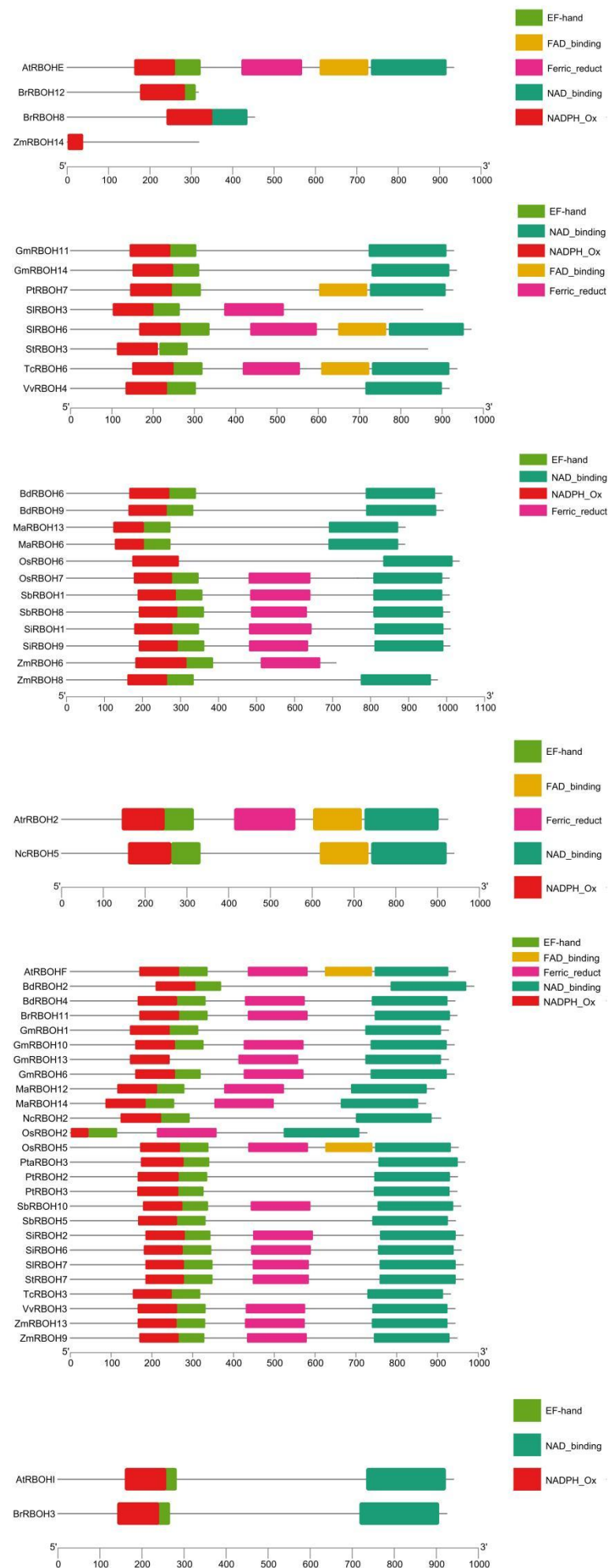

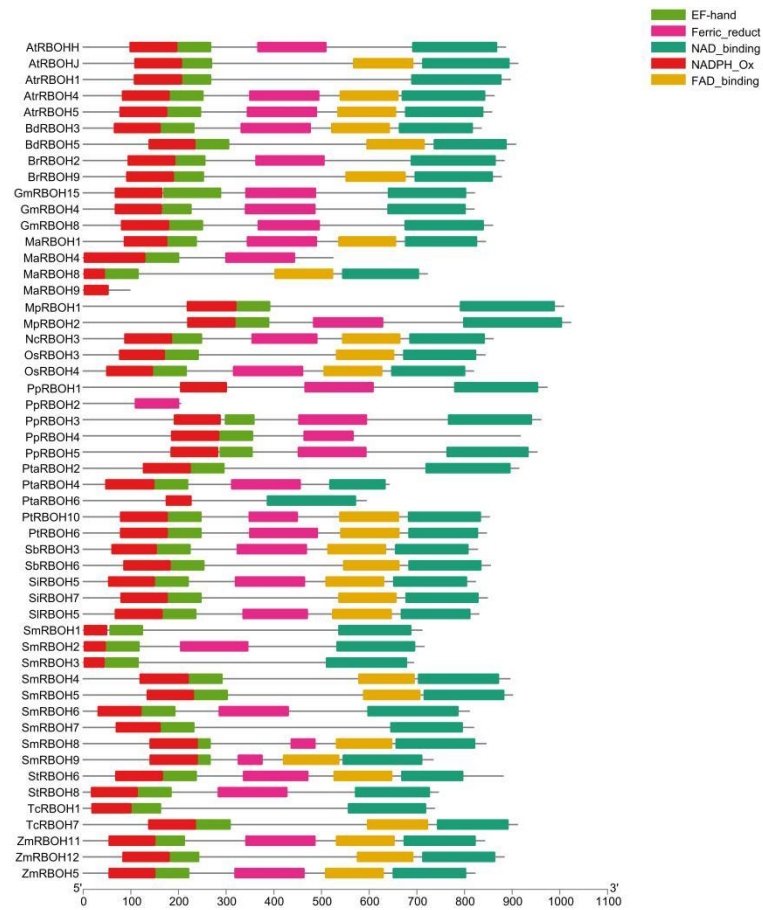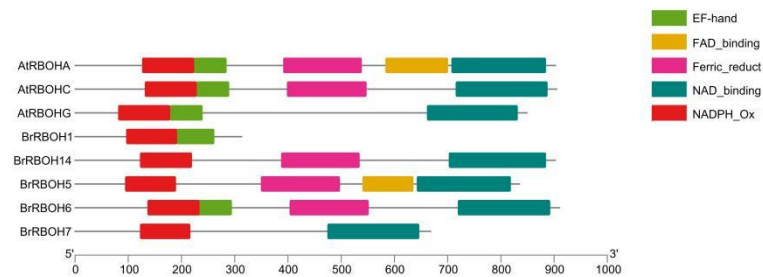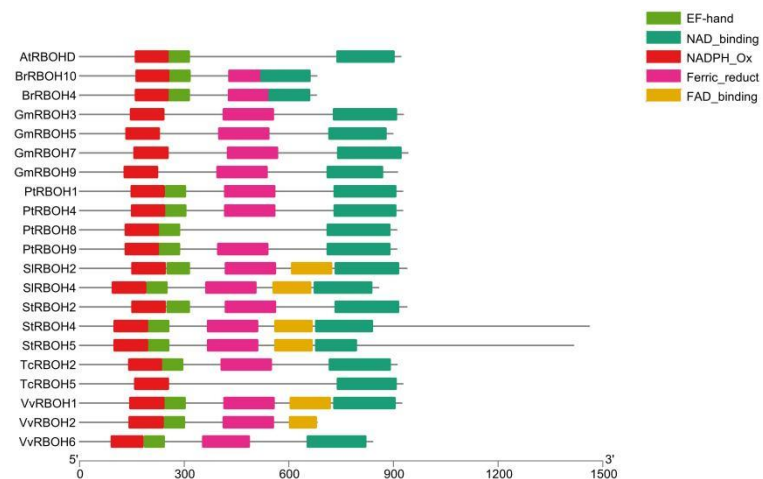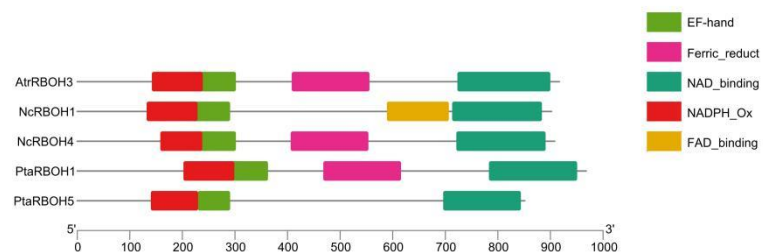

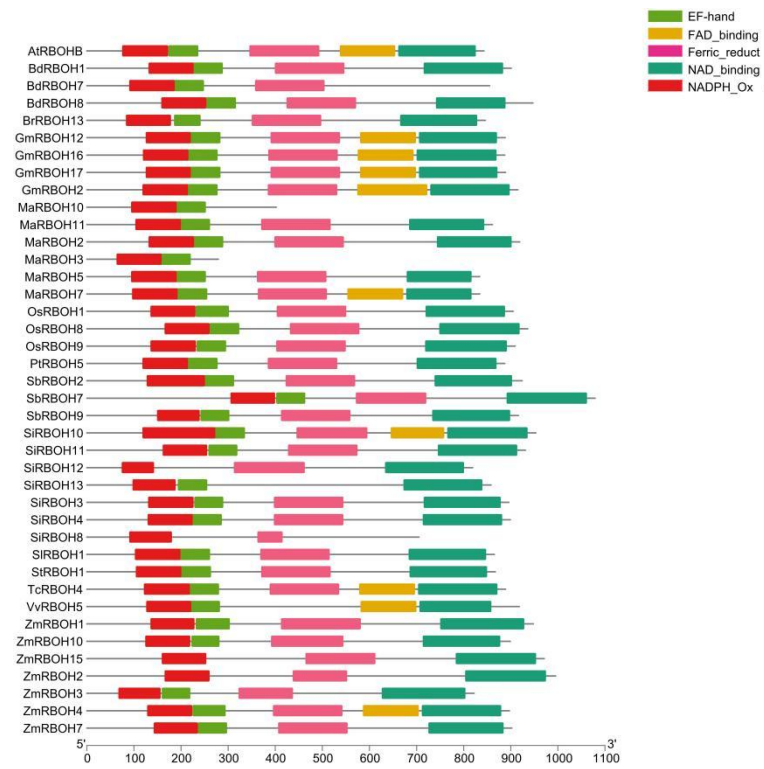

Supplement: Supplementary file 1 [file ijms-24-03858-s001.zip › Supplementary Figure S1.pdf]

**Figure S2.** Heatmaps of RBOH genes in different species in each of the five subgroups.

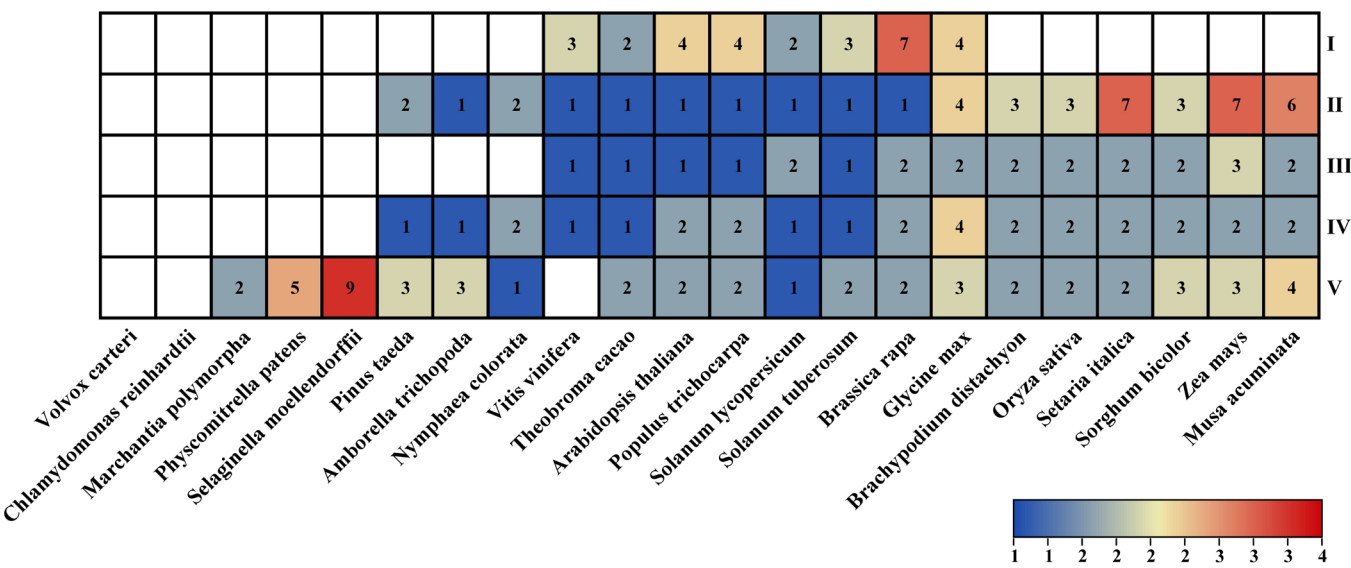

Supplement: Supplementary file 1 [file ijms-24-03858-s001.zip › Supplementary Figure S2.pdf]

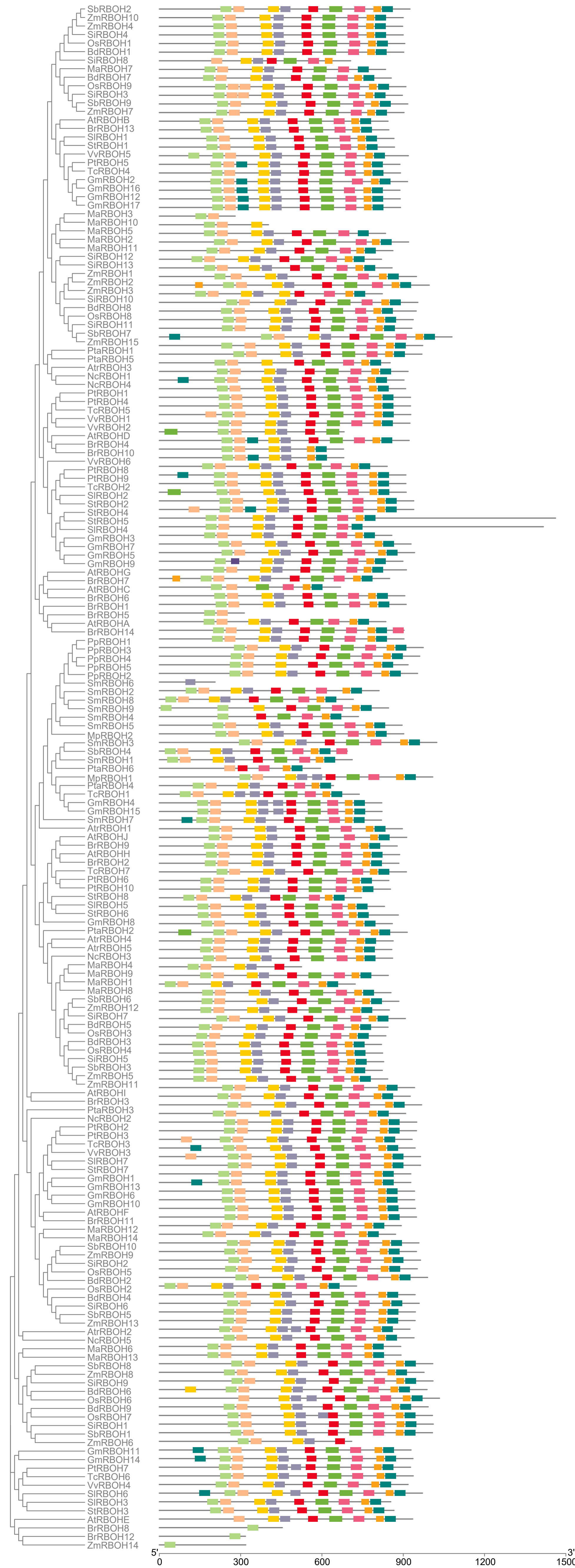

Figure S3. Conserved motifs in RBOH proteins of 20 species.

Supplement: Supplementary file 1 [file ijms-24-03858-s001.zip › Supplementary Figure S3.pdf]

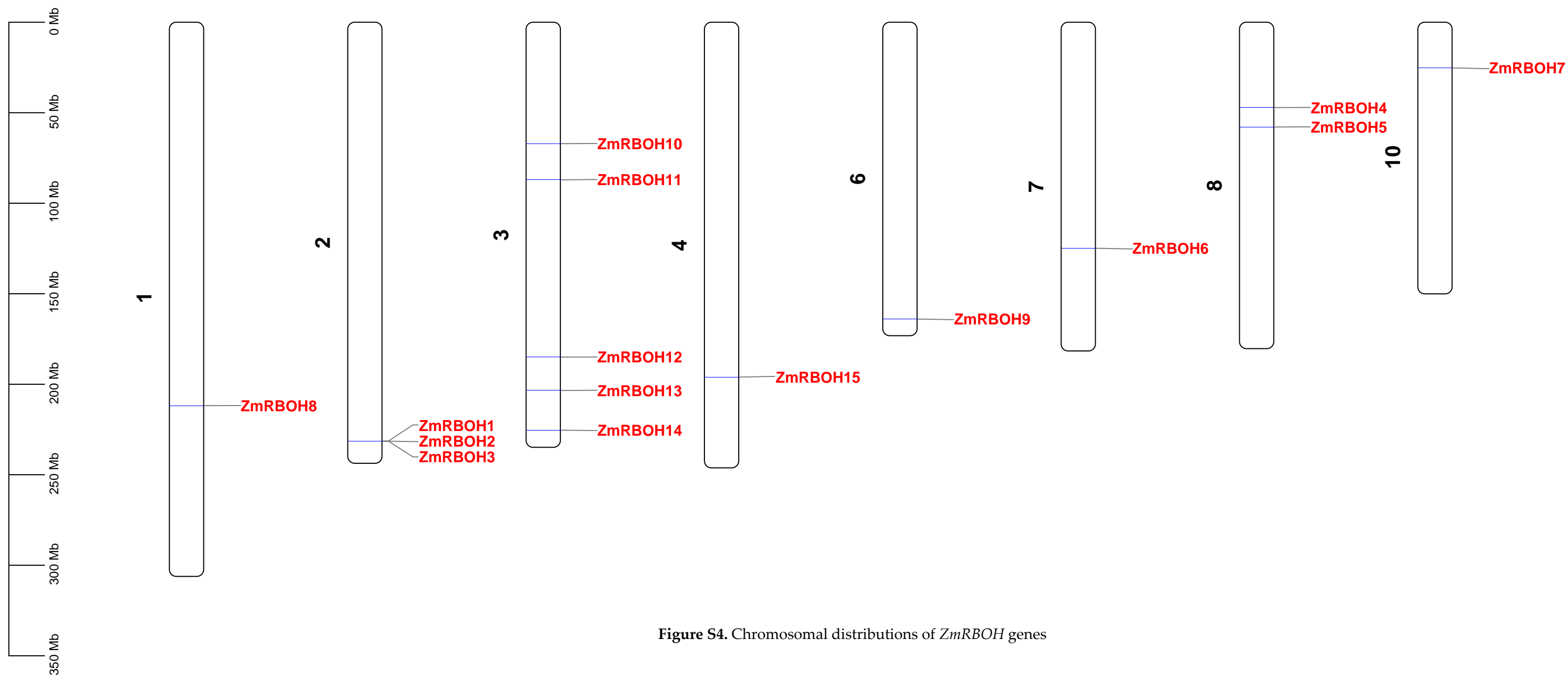

**Figure S4.** Chromosomal distributions of *ZmRBOH* genes

Supplement: Supplementary file 1 [file ijms-24-03858-s001.zip › Supplementary Figure S4.pdf]

**Figure S5.** *Cis*-elements in the promoters of *ZmRBOH* genes.

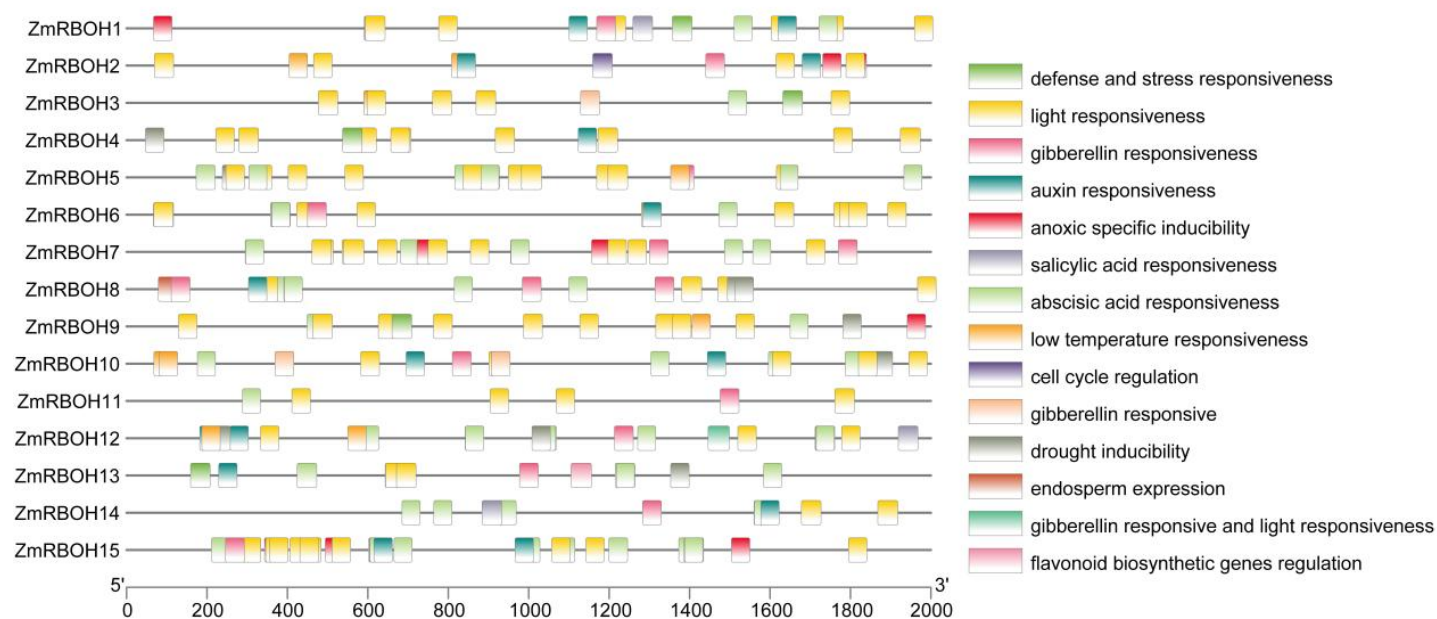

Supplement: Supplementary file 1 [file ijms-24-03858-s001.zip › Supplementary Figure S5.pdf]

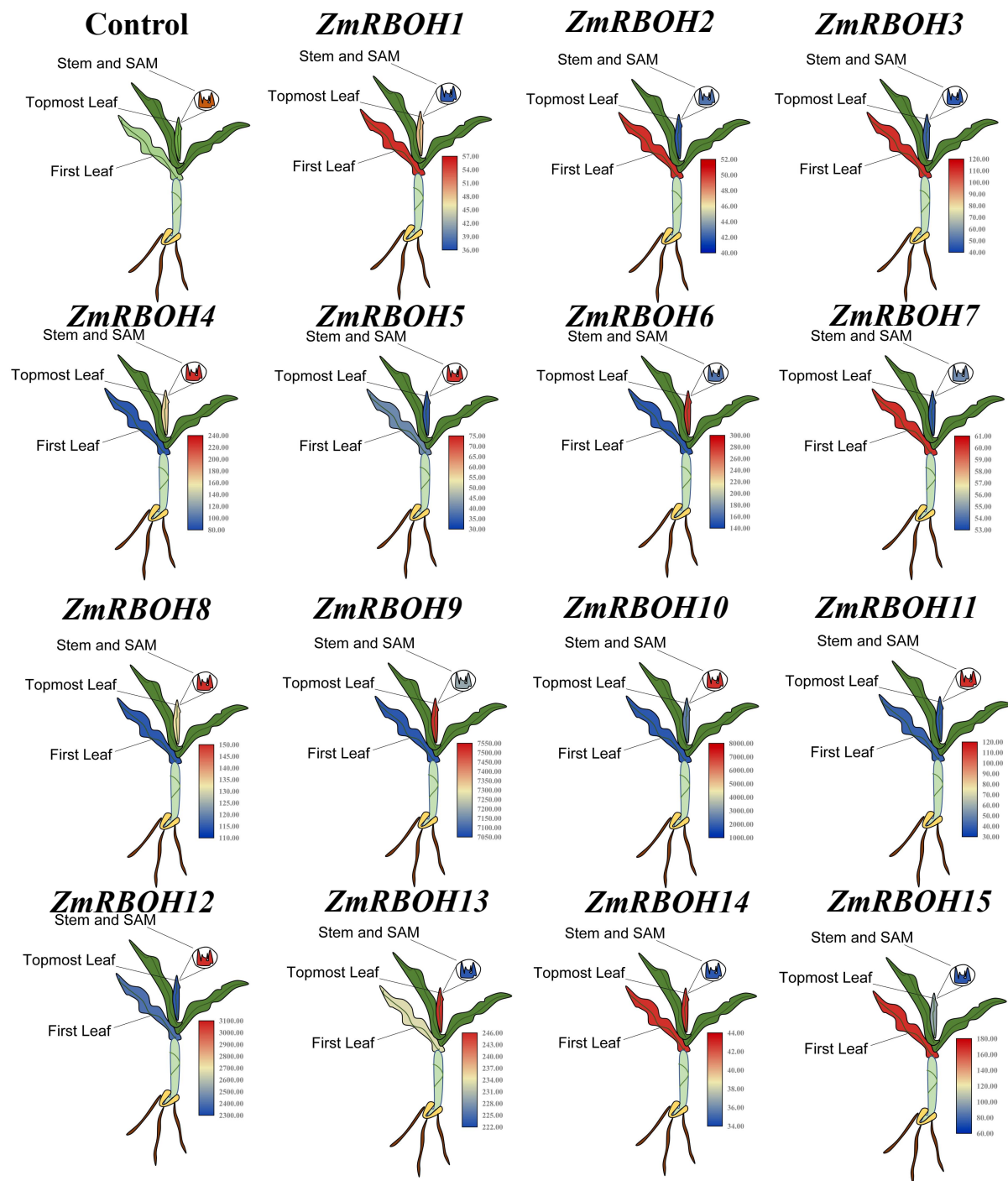

**Figure S6.** Heatmaps of phenotype simulation of *ZmRBOH* genes in V2 stage of maize.

Supplement: Supplementary file 1 [file ijms-24-03858-s001.zip › Supplementary Figure S6.pdf]
